# Rheumatoid Arthritis With Focal Segmental Glomerulosclerosis: A Case Report and Literature Review

**DOI:** 10.7759/cureus.37161

**Published:** 2023-04-05

**Authors:** Maram Albandak, Mohammed Ayyad, Samah Abu Ajamia, Ahmad Quntar, Layth Al-Karaja, Hamza M Alsaid, Laith Alamlih

**Affiliations:** 1 Internal Medicine, Al-Quds University, Jerusalem, PSE; 2 Internal Medicine, Hadassah University Hospital, Jerusalem, ISR; 3 Rheumatology, Hebron University, Hebron, PSE

**Keywords:** autoimmune disease, rheumatoid arthritis, proteinuria, nephrotic syndrome, focal segmental glomerulosclerosis (fsgs)

## Abstract

Rheumatoid arthritis (RA) is a chronic systemic autoimmune disease primarily affecting the joints and, to a lesser extent, other systems. Renal involvement in RA is rare and might be due to the presence of systemic inflammation or the toxic effect of the medications used. Of the many types of renal diseases that can affect RA patients, focal segmental glomerulosclerosis (FSGS) is rarely encountered. In this report, we present a rare co-existence of RA and FSGS in a 50-year-old female with RA who was found to have FSGS as a possible cause of proteinuria and an extraarticular manifestation of RA. The patient’s RA started as palindromic rheumatism, which progressed later to chronic symmetrical polyarthritis of the small and large joints. Along with the flare of her joint disease, she was found to have lower limb edema. Her workup showed persistent proteinuria of more than one gram per day. Renal biopsy showed unexpected findings of FSGS. Our patient was treated with tapering doses of steroids, methotrexate, candesartan, and a diuretic that controlled joint disease, blood pressure, and proteinuria. Follow-up at two years showed normal kidney function tests, a significant decline in proteinuria, and controlled joint disease. Our case portrays a possible relationship between FSGS as a cause of proteinuria in patients with RA. Physicians should be aware of the possibility of FSGS in RA patients, which can affect the management plan, medication efficacy, and overall prognosis.

## Introduction

Rheumatoid arthritis (RA) is a chronic systemic autoimmune disease primarily affecting the joints [1]. To a lesser extent, RA affects other systems such as cardiovascular, pulmonary, ocular, nervous, skin, renal, and gastrointestinal [2]. Renal involvement in RA patients is rare, and the pathogenesis is still not well understood. Mesangial glomerulonephritis and membranous glomerulonephritis are the most frequent forms of renal involvement in RA, according to the most recent cohorts exceeding the incidence of the historical RA renal finding, amyloidosis [3,4].

Focal segmental glomerulosclerosis (FSGS) is one of the most common glomerular diseases in the general population [5]. Despite that, it is rarely encountered in patients with RA [6]. The disease is characterized by the presence of sclerosis affecting parts, i.e. segmental of at least one glomerulus, i.e. focal in the entire kidney biopsy specimen when examined by microscopy or immunofluorescence. It is a histologic lesion rather than a specific disease entity resulting in various degrees of proteinuria [7]. In the general population, FSGS is frequently encountered as a cause of nephrotic syndrome which progresses to end-stage renal disease in 30%-70% of cases [8].

In this report, we present a case of unusual concurrent findings of RA and FSGS in a 50-year-old patient who presented with symptoms of hypoalbuminemia with a more than 20-year history of arthritis. We also describe all the previously reported cases in the literature, including their presentation, lab findings, treatment, and outcomes.

## Case presentation

**Figure 1:**
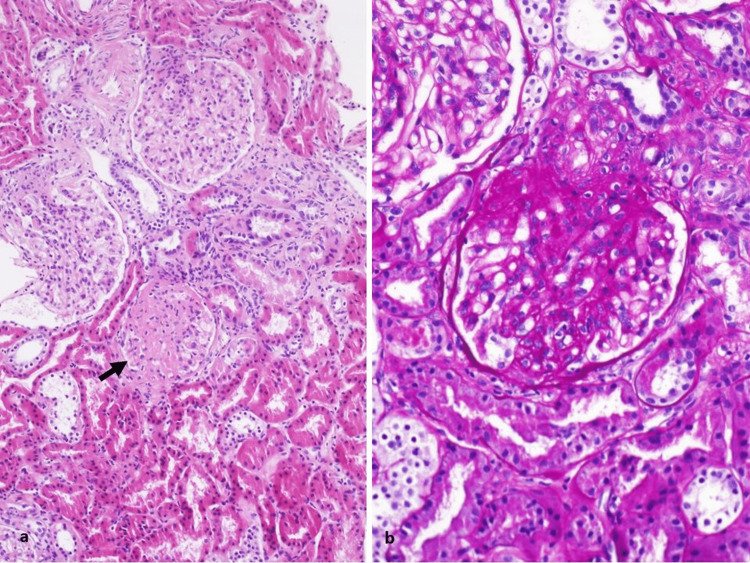
Renal biopsy with normocellular glomeruli with non-thickened glomerular basement membranes One glomerulus of 10 shows a segmental scar (arrow) consistent with focal and segmental glomerulosclerosis. (a) Hematoxylin and eosin stain, original magnification: 100; a discrete lesion of segmental sclerosis and hyalinosis is located at the glomerular vascular pole (perihilar), the glomerulus is hypertrophied. (b) Periodic acid-Schiff stain, original magnification: 200

A 50-year-old female patient presented with a history of rheumatoid arthritis (RA) since the age of 20 years. Her disease started as recurrent attacks of palindromic rheumatism for which she did not receive any treatment for 30 years. Later, the disease progressed to chronic polyarthritis of the small and large joints seven months before presenting to our clinic. The pain had inflammatory features, including significant early morning stiffness. She had also noticed bilateral lower limb edema for the last three weeks prior to her presentation to the clinic. She denied any history of mouth ulcers, photosensitivity, sicca, fever, weight loss, Raynaud’s phenomenon, skin rash, subcutaneous nodules, hair loss, or urinary symptoms. A review of the remaining systems was otherwise unremarkable. There was no history of drug abuse. She had a strong family history of arthritis in multiple first-degree relatives. Furthermore, two of her sisters were found to have proteinuria of unknown etiology. On physical examination, her blood pressure was 150/90 and her vital signs were otherwise normal. There was no previous documentation of high blood pressure readings prior to her initial visit. Her body mass index (BMI) was as high as 40. She also had multiple tender and swollen joints affecting mainly the metacarpophalangeal (MCP) joints and both knees. There was bilateral lower limb edema. After thorough clinical evaluation, laboratory investigations were ordered and showed +2 proteinuria on urine analysis with no active sediments; creatinine of 0.63 mg/dl; blood urea nitrogen (BUN) of 12.4 mg/dl; albumin of 4.29 g/dl; erythrocyte sedimentation rate (ESR) of 52 mm/hr; C-reactive protein (CRP) of 15g/l; and fasting blood sugar of 93.8 mg/dl. Her complete blood count, liver function tests, serum electrolytes, hemoglobin A1c, and complement levels were normal. Her anti-nuclear antibodies (ANA), rheumatoid factor, anti-cyclic citrullinated peptide, virology profile of hepatitis B (HBV), human immunodeficiency virus (HIV), and hepatitis C (HCV) were all negative. The 24-hour urine collection for protein was initially 1683.24 mg. Imaging studies were ordered, including a renal ultrasound, which revealed normal size and morphology of both kidneys, as well as incidental hepatomegaly with diffuse fatty echotexture. Based on her clinical and laboratory presentation, she was prescribed methotrexate 10 mg orally weekly, prednisolone 5 mg orally daily, and folic acid. Despite being compliant with her medications, she didn’t notice any significant improvement in the joint pain. Regular follow-up showed persistent proteinuria of more than one gram daily so a kidney biopsy was performed one year after her first presentation to the clinic, which revealed evidence of FSGS (Figure 1). The patient continued taking oral methotrexate and her dosage was increased to 20 mg weekly to control her rheumatological disease and prevent its progression, along with prednisolone 5 mg daily. She was followed monthly. An attempt to wean off steroids was undertaken many times but the joint pain kept flaring up with each attempt. This led us to add oral hydrochlorothiazide 25 mg daily to further control her joint disease as we tried to wean off steroids. Candesartan 8 mg once daily was also given to reduce proteinuria and protect the kidneys.

During the two-year follow-up, the patient continued to improve gradually, maintaining normal kidney function tests. Uranalysis at baseline showed +2 for protein; the patient then was treated with methotrexate and prednisolone that was eventually weaned off. Her proteinuria over the next two years continued to improve, with the dipstick being +1 in 2019 and nil by the end of 2021. At the end of the two-year follow-up, her urine dipstick was negative and her 24-hour urine protein dropped to 218 mg along with normal kidney function tests. Urine analysis showed trace protein.

## Discussion

This case is a rare example of proteinuria in a patient with RA due to FSGS. The diagnosis of RA was based on the 2010 Rheumatoid arthritis classification criteria with the presence of the clinical characteristics of RA, i.e chronic polyarthritis with the predominance of small joints particularly metacarpophalangeal MCP joints, high ESR of 52 mm/hr, and negative serology, for a duration of >6 weeks, which added up to 6 points total [9]. In patients with RA, many conditions can result in proteinuria, thus, a kidney biopsy was crucial to determine the cause, which was, in this case, FSGS. The patient’s renal disease lagged 28 years behind the start of her arthritis and presented with a worsening of her symptoms. Common associations with FSGS were excluded such as the intake of heroin, medications, including non-steroidal anti-inflammatory drugs (NSAIDs) and penicillamine, infections such as HIV and HBV, as well as other diseases such as vasculitis [10,11].

FSGS remains the most common primary glomerular pathology leading to end-stage renal disease in the United States, and its prevalence has been gradually increasing over the years [12]. FSGS can be divided into the primary (idiopathic) and secondary subtypes. Secondary causes of FSGS include medications, infections, drugs of abuse, and connective tissue disorders [12]. Although rare, long-term medical management of RA could be a possible cause of renal impairment, as we alluded earlier, our patient was diagnosed with palindromic RA in her 20s and she did not require medical management to control her symptoms till her recent current flare that was already associated with proteinuria. This basically excludes medical management of RA as a possible cause for her FSGS.

The patient was treated with methotrexate and angiotensin-converting enzyme (ACE) inhibitors with good control of her joint and kidney disease. The emergence of FSGS while the patient is having active joint disease, and its resolution after controlling it with immunosuppressive agents, might indicate a possible association between active RA flares and FSGS renal disease. Somehow, the two diseases have similar immunopathophysiologies [10,13]. Another possible risk factor for FSGS in this patient is the obesity and insulin resistance evidenced by her high BMI and fatty liver. However, the temporal association between RA flare-ups and proteinuria makes FSGS due to this medical condition a more plausible explanation.

The pathogenesis of RA is complex with many factors contributing to the disease, including genetic, environmental, and immunologic. The disease occurs mainly due to the deposition of complexes in many organs particularly the joints. To a lesser extent, deposition might affect the kidneys as well, leading to glomerular basement membrane damage resulting in increased protein permeability. This is aggravated by T-cell mediated damage and cytokine production, which is also a hallmark pathway behind FSGS pathogenesis and could indicate possible crosstalk between FSGS and RA on the cellular and immunological level [10,13].

The initial treatment of FSGS, whether primary or secondary, is the optimization of blood pressure control with the use of ACE inhibitors or angiotensin II receptor blockers (ARBs) [14]. Patients with primary FSGS who are persistently nephrotic after a course of conservative management, or patients who present with complications of nephrotic syndrome, require more aggressive treatment with prednisone or other immunosuppressive regimens [15,16]. In contrast, in secondary forms of FSGS, the mainstay of treatment is blood pressure control, ACE inhibitors, ARBs, and disease-specific treatment if available, e.g. HIV treatment [14]. Our treatment of the patient had features of both: conservative management with blood pressure control, and disease-targeted therapy against RA, which was generally immunosuppressive.

Renal involvement in RA might be due to the presence of systemic inflammation or the toxic effect of the medications. It is noted that extra-articular involvement in RA was reduced significantly in the last decade, which could be due to the development of new potent medications [17]. An example of that is the reduction in the development of amyloidosis in patients with RA [3,18]. Although severe renal impairment in RA is rare, it was found that RA patients have a higher incidence of reduced kidney function as compared to non-RA subjects, particularly in patients with NSAID use and higher inflammatory markers [19]. Proteinuria affects 3-7% of patients with RA in general [20]. However, in RA patients who are hospitalized, significant proteinuria can be found in up to 50% [21]. There is scarce data about the occurrence of FSGS in the RA cohorts. However, some reports indicate a higher prevalence in Chinese and Egyptian populations as compared to Hispanic [3,4,22].

Most newly diagnosed FSGS cases are either secondary or due to an undiagnosed genetic cause rather than a primary type [5]. Interestingly, two first-degree family members of the patient had a history of arthritis and proteinuria. The presence of unexplained proteinuria in her sisters might indicate that the coexistence of diseases is due to a genetic factor. A recent report showed a similar association when it described a mutation in the NPHS2 gene in a patient with rheumatoid factor positive juvenile idiopathic arthritis (JIA) and proteinuria who has two siblings affected with FSGS [23]. Indeed, this association still needs further evaluation.

We reviewed the literature looking for similar cases, searching the Medline and Scopus databases with no restrictions. We found six cases, including ours, as summarized in Table 1 [10,11,13,24,25]. Most patients were females, consistent with RA gender distribution. Patients were from variable age groups and ethnicities. Interestingly the RA disease was either active on the diagnosis of FSGS or very severe, resulting in deformity, which might indicate the role of RA disease activity as a possible cause of this association. This is also supported by the fact that all RA cases were diagnosed with the disease years before FSGS, indicating that RA or RA medications are the cause behind FSGS. The range of proteinuria was wide, reaching up to 37 grams per day [11]. Inflammatory markers were high in most patients, suggesting active rheumatoid disease. Most patients were found to have positive serology for rheumatoid and two patients were found to have positive ANA as well [11,25]. All patients but one responded to immunosuppressive treatment and ACE with a very favorable response. Only one patient was found to have raised kidney function tests who later developed ESRD, requiring dialysis [25].

**Table 1:** Summary of the major characteristics of patients with RA and associated FSGS NM= Not mentioned, ATN= Acute Tubular Necrosis, FSGS= Focal Segmental Glomerulosclerosis, NS= Nephrotic Syndrome, +ve= Positive, -ve= Negative, MH= Microscopic Hematuria, PU= Proteinuria, ESRD= End stage renal disease, RF=Rheumatoid factor, ANA=Anti-nuclear antibody, ENA =extractable nuclear antigen, CCP=cyclic citrullinated peptide, PANCA=Perinuclear Anti-Neutrophil Cytoplasmic Antibodies, Ds=Double-stranded, KFT=Kidney function test, JIA=Juvenile idiopathic arthritis

Case/year	Our case 2021	Liu et al. 2017 [10]	Madan et al. 2015 [24]	Varma et al. 2010 [13]	Gedalia et al. 2001 [25]	Sugiyama et al. 1997 [11]
Age at FSGS diagnosis	50	54	60	17	14	45
Sex	Female	Male	Female	Female	Female	Female
Country/ Ethnicity	Palestine	China	USA	India/NM	African American	Japan
Obesity	Obese	NM	Obese	NM	No	No
RA duration since FSGS diagnosis	28 years	8 years	NM	3 years	1 year	7 years
RA severity/activity	Active disease at the diagnosis	Active disease when found to have FSGS	Deforming disease but controlled upon presenting with edema	Active disease at diagnosis	Active at the diagnosis	RA was in remission
Medications taken before/at diagnosis	Corticosteroids Methotrexate	Corticosteroids, NSAIDs (loxoprofen, celecoxib), Methotrexate, Leflunomide	NM	Indomethacin prednisolone (10 mg) hydroxychloroquine	Oral low-dose methotrexate (12.5 mg) once a week	NSAID, Prednisolone 10mg, Penicillamine 100mg
severity of Proteinuria	>1 gram per 24h	12 grams per 24h	2.5 grams per 24 hours	9 grams per 24 hours	2+ protein	37 g per 24h
Hematuria	No	No	NM	No	Yes	No
Raised renal function tests at presentation	No	No	No	Yes	No	No
ESRD	No	No	No	No	No	No
Histopathology	Light microscopy: One glomerulus of ten shows a segmental scar associated with mild interstitial fibrosis. Electron microscopy: no dense deposits. Fusion of foot process. Immunofluorescence: negative.	Light microscopy: glomeruli with segmental sclerosing lesions. Electron microscope: no dense deposits. Visceral epithelial cell foot process effacement. Immunofluorescence: negative.	Light microscopy: glomerular scarring; Intracapillary hyaline dense deposits were identified in sclerotic segments. Electron microscopy: no dense deposits. Immunofluorescence: negative.	Light microscopy and Electron microscopy: Two of 25 glomeruli showed segmental sclerosis with normal tubules, interstitium, and small arterioles. Immunofluorescence: scanty mesangial C3 deposits.	Light microscopy: Frequent globally sclerotic glomeruli; glomeruli with focal segmental glomerulosclerosis; Moderate interstitial fibrosis; Moderate tubular atrophy. Electron microscopy: No deposits; segmental sclerosis with corrugation of the glomerular basement membrane; Effacement of the foot processes. Immunofluorescence: negative	Light microscopy: Two of 15 glomeruli in the deep cortex showed segmental hardening with hyalinosis and foam cells. Electron microscopy: dense deposits in a glomerulus with segmental sclerosis. Immunofluorescence: segmental IgM deposits.
Inflammatory markers upon diagnosis	ESR 52 mm/h CRP 15 mg/L	ESR 88 mm/h, CRP 18.8 mg/L	Raised CRP	ESR 102 mm/h	ESR 127 mm/h	ESR 85 mm/h
Serology (RF, ANA, ENA, antiCCP)	RF -ve ANA -ve Anti-CCP -ve	RF +ve Anti CCP +ve ANA1:80 ENA: -ve	NM	RF +ve ANA -ve Ds-DNA-ve	RF +ve ANA 1:320 PANCA +ve ENA -ve	RF +ve ANA +ve
Treatment	Prednisolone Methotrexate candesartan and hydrochlorothiazide	IV Methylprednisolone followed with prednisone oral steroids. Celecoxib200mg/BID, leflunomide10mg/QD.	Steroids and ACEI	High-dose prednisolone (60 mg/day). Methotrexate (12.5 mg/week). Hydroxychloroquine therapy angiotensin II receptor blockers, statins spironolactone	Methotrexate which was stopped later. Prednisone 60 mg/day Azathioprine 75 mg/day. Monthly IV cyclophosphamide Pulse therapy.	Prednisolone 60mg/ day
Follow-up and Outcome	Decline in proteinuria to 218 mg per day with normal kidney function tests.	Decline in proteinuria and normal KFTs	After 8 months, FSGS relapsed which was associated with RA flares. Later, proteinuria decreased to about 500mg per day.	Significant improvement of 24-h proteinuria with normal renal function tests. Overall, a favorable response to disease-modifying drugs for JIA and high-dose steroids.	Improvement of arthritis; Progressive worsening of the renal function leading to ESRD	Significant improvement with high-dose steroids

It is crucial to identify the presence of FSGS in RA patients to give proper treatment and follow-up. In fact, it was found that the elimination half-life of adalimumab is significantly shorter in patients with FSGS [26]. This will affect the response of patients to adalimumab, which is one of the crucial disease-modifying RA medications. It is possible that FSGS might affect other rheumatoid medications but further research is needed to elaborate on that. In our case, methotrexate was used to control the symptomatic joint disease without an obvious decline in kidney function, while high-dose steroids were used to treat joint disease as well as proteinuria, which was suspected to be due to secondary FSGS.

It is worthy to note that our patient was treated during the coronavirus disease 2019 (COVID-19) outbreak. Severe infection with COVID-19 can evoke symptomatic FSGS, non-collapsing type, especially in black patients, and it is often associated with the presence of high-risk alleles and the presence of risk factors for kidney disease. Despite that, our patient developed mild COVID a few months after her initial flare of proteinuria and did not require any treatment or hospitalization. FSGS in our patient was discovered with her flares of RA, unrelated to the time of her COVID infection, which makes COVID-19 less likely the culprit of her nephrotic syndrome compared to her RA diagnosis of 30 years [27].

## Conclusions

In conclusion, FSGS can rarely co-exist in patients with RA either directly or indirectly linked to underlying arthritis. Immunosuppressive therapy and ACE inhibitors are the mainstays of treatment. We believe that the early FSGS diagnosis along with appropriate management measures, including early treatment with steroid-sparing agents and ACE inhibitors can aid in decreasing morbidity and decrease the need for NSAIDs. Hence, prompt referral to nephrology should be sought for all patients who present with urinary symptoms and proteinuria. We hope that this report can help future studies to better characterize this possible rare disease association and provide further information and guidance regarding its prognosis and management.
